# Effects of Extruded Sugar Beet Pulp By-Product on Bioactive Composition, Antioxidant Activity, and Quality of Classic and High-Protein Gluten-Free Pasta

**DOI:** 10.3390/molecules31152650

**Published:** 2026-07-29

**Authors:** Marek Kruczek, Dorota Gumul, Karolina Miśkiewicz, Justyna Rosicka-Kaczmarek, Gabriela Cedzyńska, Karolina Pająk, Katarzyna Szary-Sworst

**Affiliations:** 1 Department of Carbohydrate Technology and Cereal Processing, Faculty of Food Technology, University of Agriculture in Krakow, 122 Balicka Street, 30-149 Krakow, Poland; gabriela.cedzynska@student.urk.edu.pl (G.C.); karolina.pajak@student.urk.edu.pl (K.P.); katarzyna.szary-sworst@urk.edu.pl (K.S.-S.); 2 Institute of Food Technology and Analysis, Faculty of Biotechnology and Food Sciences, Lodz University of Technology, 2/22 Stefanowskiego Street, 90-537 Lodz, Poland; karolina.miskiewicz@p.lodz.pl (K.M.); justyna.rosicka-kaczmarek@p.lodz.pl (J.R.-K.)

**Keywords:** antioxidant activity, bioactive compounds (polyphenols), extruded sugar beet pulp, gluten-free pasta, high-protein gluten-free pasta, quality

## Abstract

This study investigated pure extruded sugar beet pulp (ESBP), a by-product containing 58.0% dietary fiber, 6.2% protein, and 18.0% sugars as a novel functional ingredient for conventional and high-protein (HP) gluten-free pasta. The analysis of cooking quality showed that cooking losses increased with enrichment, reaching a 2.9-fold increase in the HP_15% variant. In terms of texture, ESBP addition increased cutting work to 9.65 N·s in HP_15%, suggesting improved matrix cohesion while maintaining stable instrumental firmness. Enrichment also caused noticeable darkening; in the conventional system, lightness (L*) decreased from 70.8 to 56.2 after cooking at the 15% addition level. Although 15% ESBP resulted in the greatest bioactive enrichment, including a 72% increase in phenolic content and significantly enhanced ABTS antioxidant activity, HPLC-DAD analysis confirmed that the phenolic profile was dominated by hydroxybenzoic acids, particularly gallic and 4-hydroxybenzoic acids, whose contents increased by up to 48% and 44%, respectively. Consumer acceptance testing revealed that ESBP levels above 10% markedly reduced acceptability, including a 35% lower overall acceptability score in the HP system, due to a pronounced earthy aroma and excessive cooking losses. The results indicate that ESBP functionality is highly matrix-dependent and that a 10% substitution level represents the optimal compromise between improved bioactive properties and acceptable technological and sensory quality. These findings highlight the potential of pure ESBP for the development of specialized foods with high nutritional and health-promoting value, while future research should focus on the gastrointestinal bioaccessibility of phenolic compounds and overcoming the technological limitations associated with their use.

## 1. Introduction

Pasta is one of the most widely consumed cereal products worldwide due to its high energy value, ease of preparation, and broad culinary applications [1,2,3,4]. According to data from Statistics Poland (GUS) for 2018, the average Pole consumes approximately 5.6 kg of pasta annually. Compared with 2017, this represented an increase in consumption of as much as 21.7% [5]. The dynamic growth of this sector is driven, among other factors, by changing market conditions and increased demand for foods with extended shelf life [6]. At the same time, a growing interest in gluten-free foods can be observed worldwide. Forecasts indicate that the global gluten-free products market will grow at an annual rate of 10.1% between 2026 and 2033 [7]. This trend results not only from the medical needs of individuals with celiac disease but also from increasing consumer interest in gluten-free products, which are often perceived as a healthier alternative to conventional foods, although this belief is generally not supported by medical evidence [8,9]. It is estimated that celiac disease affects between 0.7% and 1.4% of the global population [10]. The number of diagnosed cases continues to increase due to improvements in diagnostic methods and greater awareness of disease symptoms. Currently, lifelong adherence to a strict gluten-free diet remains the only effective treatment for this autoimmune disorder [11]. These factors highlight the need for further development of the gluten-free food sector, particularly in terms of improving the nutritional, functional, and technological quality of products. Despite their market expansion, gluten-free products still face significant nutritional and technological limitations. They are frequently characterized by low protein content and high levels of simple carbohydrates, while the absence of glutenins and gliadins poses a major challenge in stabilizing the starch matrix, leading to increased brittleness and reduced structural integrity. Since many gluten-free products are based primarily on starches, including rice, corn, and potato starch, they often exhibit low nutrient density and a high glycemic index. In the case of gluten-free pasta, the glycemic index may range from approximately 60 to 70, whereas traditional durum wheat pasta typically exhibits lower values of 45–55 [12]. In addition, compared with conventional wheat products, gluten-free foods are often more highly processed and may contain greater amounts of fats and simple carbohydrates [13,14]. Excessive consumption of such products may contribute to obesity, cardiovascular diseases, and metabolic disorders [7,15]. Analyses have shown that gluten-free pasta contains, on average, 6–10 g of protein per 100 g of product, whereas durum wheat pasta usually provides 11–15 g of protein per 100 g [14]. A major technological challenge in gluten-free pasta production is the absence of the gluten network formed by glutenins and gliadins in traditional wheat products. The lack of this network makes starch matrix stabilization more difficult, negatively affecting the structure and quality of the final product [16,17]. Consequently, gluten-free pasta may exhibit poorer textural properties, greater brittleness, faster drying, and excessive cooking losses due to the leaching of solids into the cooking water. To address these challenges, the functional food sector is increasingly exploring plant-based ingredients that could improve both the nutritional value and technological properties of gluten-free products [18]. Previous studies have investigated the fortification of gluten-free pasta with various raw materials, including tomato pomace, flaxseed flour, almond press cake, carrot pomace, fish powder, and algae [7,18,19,20]. One promising approach involves the use of by-products from the sugar industry. The European Union is one of the global leaders in sugar beet production, producing approximately 15.5 million tons of sugar annually, which accounts for around 50% of global sugar beet sugar production [21]. However, this process generates substantial amounts of by-products. Sugar beet pulp and molasses are estimated to represent 20–40% of the processed beet mass [22]. Producing 9 tons of sugar requires harvesting approximately 63 tons of sugar beets per hectare (European Commission), which is associated with a considerable environmental burden, including the use of 5500–7500 m^3^ of water per hectare of cultivation. In line with the principles of the circular economy and the European Union’s “zero waste” action plan, efforts are being made to improve resource efficiency and reduce food waste, which currently accounts for approximately 20% of food production within the EU [23,24,25]. Sugar beet pulp is a valuable by-product with considerable potential for application in the food industry [26,27]. It is characterized by a very high dietary fiber content of approximately 60–70 g/100 g dry matter. This fiber consists mainly of cellulose, hemicelluloses, and pectins, with only a small proportion of lignin. The insoluble fiber fraction predominates over the soluble fraction, where pectin fraction differs from citrus and apple pectins due to its high degree of acetylation and feruloylation [28]. In addition to dietary fiber, sugar beet pulp contains polyphenols with antioxidant properties, including phenolic acids such as ferulic and vanillic acids [28,29]. Among these compounds, ferulic acid is of particular technological importance, as it may account for approximately 1% of sugar beet pulp mass and act as a natural cross-linking agent. By promoting the formation of stable gel-like structures, ferulic acid may contribute to strengthening the gluten-free pasta matrix and reducing the leaching of solids into the cooking water [28]. The use of extruded sugar beet pulp may further enhance its technological suitability. The extrusion process can reduce the characteristic flavor of this by-product, which is partly associated with the presence of geosmin [30]. Furthermore, extrusion loosens the raw material structure and increases its water-binding capacity [31,32], which may positively affect the texture of gluten-free products. Additionally, the extrusion process may cause the disruption of covalent and non-covalent bonds between carbohydrates and proteins, releasing smaller molecular fragments that can more readily interact with starch molecules. As a result, extruded sugar beet pulp (ESBP) components, particularly arabinan and pectin fractions, may partially compensate for the absence of gluten by contributing to the formation of a cohesive network that stabilizes the starch matrix, thereby directly contributing to improved textural and cooking properties of the final product [28]. Therefore, due to its high fiber content and the presence of bioactive compounds, sugar beet pulp may serve as an ingredient that improves both the nutritional value and technological properties of gluten-free pasta. Previous studies, including those by Simić et al., 2021 [32], have focused on the use of a co-extruded mixture of corn grits and sugar beet pulp, in ratios of 85:15, 70:30, and 55:45, as a base for cookie production. However, the present study represents the first approach to using pure extruded sugar beet pulp, produced exclusively from sugar beet pulp with a moisture content of approximately 20% and without starch-based additives during the extrusion stage, as a direct functional ingredient. To the best of the authors’ knowledge, this is the first study to investigate the potential of ESBP in a gluten-free pasta matrix and the first comprehensive comparison of the effect of this additive on the technological properties and bioactive profile of two distinct systems: conventional and high-protein gluten-free pasta. Another approach to improving the nutritional quality of gluten-free pasta is protein enrichment through the incorporation of plant protein sources, such as pea protein isolate. This strategy allows the development of products with higher protein content and a more favorable amino acid profile, which may be particularly beneficial for physically active individuals and consumers seeking foods with enhanced nutritional value [33].

The aim of this study was to evaluate the potential of extruded sugar beet pulp (ESBP) as a functional ingredient in the development of two types of gluten-free pasta. The study compared conventional and high-protein gluten-free pasta formulations by assessing their physicochemical characteristics, technological quality, cooking performance and consumer acceptance, and also polyphenols determined by spectrophotometric methods, selected phenolic compounds determined by HPLC and antioxidant capacity.

## 2. Results and Discussion

### 2.1. Characteristics of Extruded Sugar Beet Pulp (ESBP) as an Ingredient for the Enrichment of Gluten-Free Pasta

**Figure 1:**
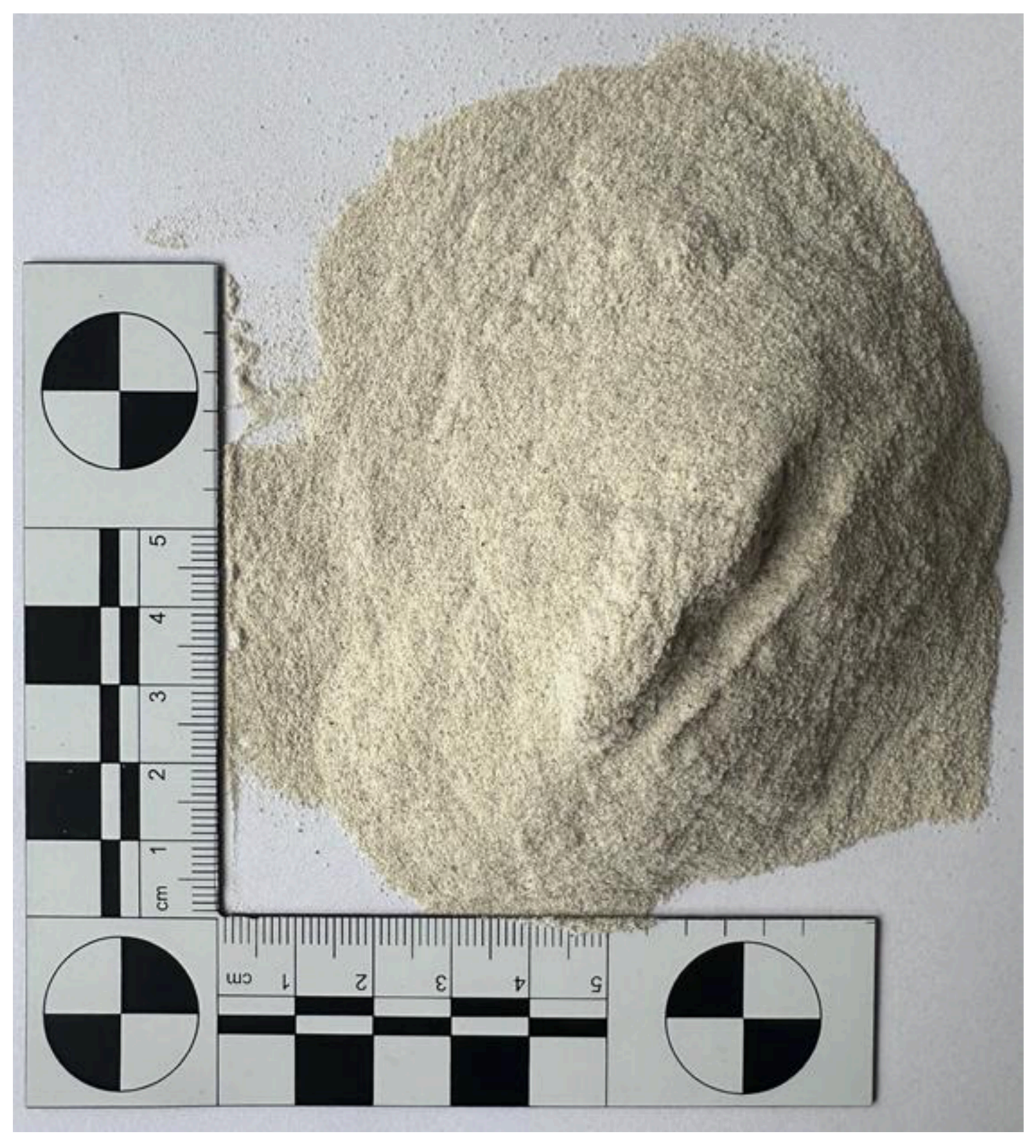
Extruded sugar beet pulp (ESBP).

**Figure 2:**
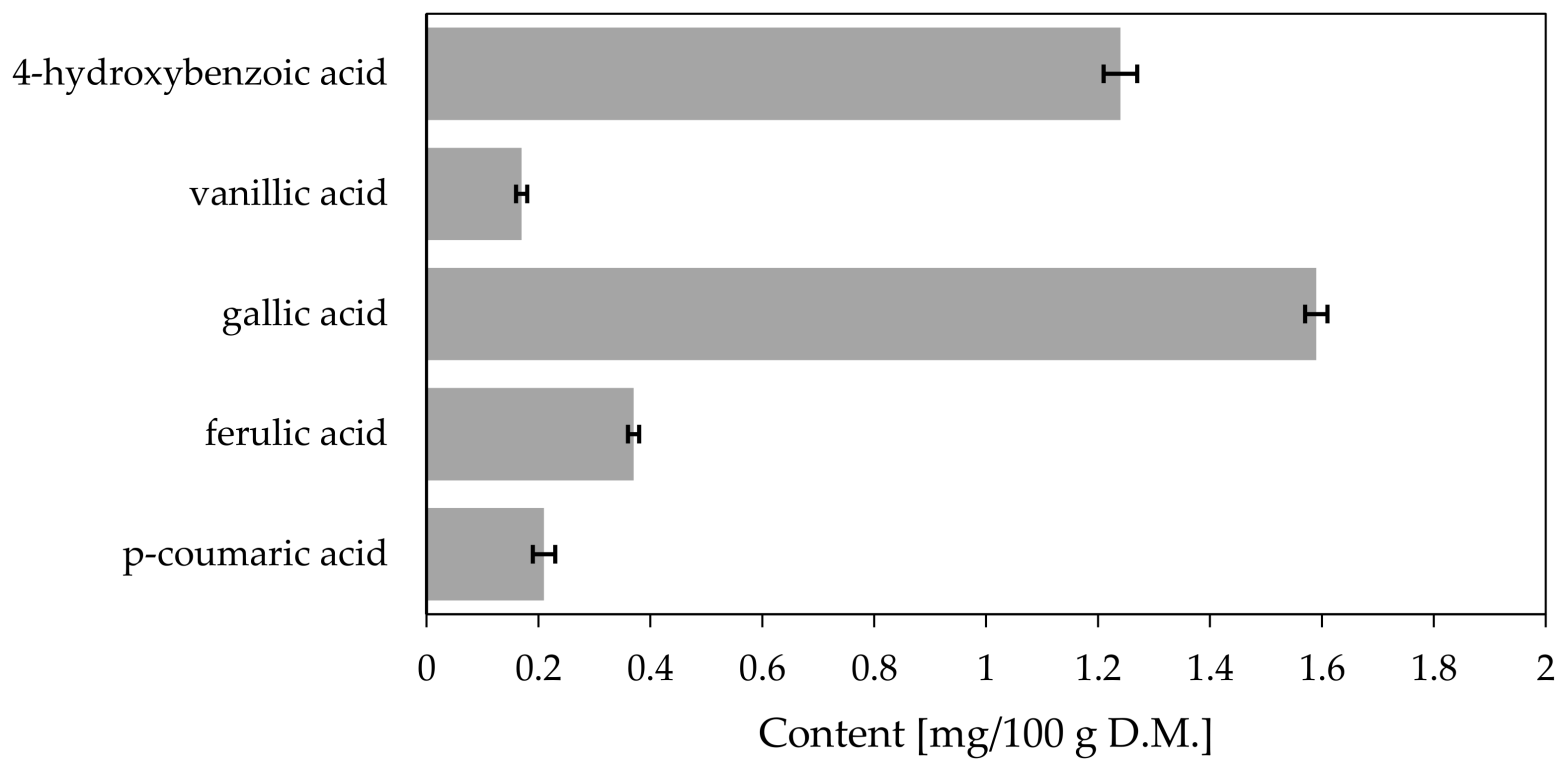
Individual phenolic compounds determined by HPLC in extruded sugar beet pulp.

Sugar beet pulp is a by-product of the sugar industry, obtained after the juice is extracted from shredded sugar beet roots (Figure 1). It is a microbiologically unstable material, prone to rapid spoilage and characterized by a strong earthy aroma. Therefore, extrusion may be an interesting alternative to traditional drying methods, as it enables both loosening of the pulp structure and reduction of the earthy aroma. The research material consisted of extruded sugar beet pulp (ESBP), which was analyzed in terms of its nutritional composition (Table 1). ESBP was characterized by a high content of dietary fiber and carbohydrates, including sugars, a moderately low protein content, and a very low fat content. The energy value of this by-product was 310 kcal per 100 g of product (Table 1). The phenolic profile confirmed the presence of phenolic acids, flavonols, and flavonoids, whereas anthocyanins were not detected. The antioxidant activity assessed using DPPH and ABTS assays further confirmed the antioxidant potential of this by-product (Table 1). HPLC analysis showed that the identified phenolic acid profile of ESBP was dominated by hydroxybenzoic acids, with a total content of 3.00 mg/100 g (Figure 2). Among this group, gallic acid was the predominant compound (1.59 mg/100 g D.M.), followed by 4-hydroxybenzoic acid (1.24 mg/100 g D.M.) and vanillic acid (0.17/100 g D.M.). Hydroxycinnamic acids were detected at lower levels, with ferulic acid and p-coumaric acid reaching 0.37 and 0.21 mg/100 g D.M., respectively, giving a total of 0.58 mg/100 g D.M. for this group (Figure 2). The phenolic composition observed in this study generally agrees with previous reports on sugar beet and materials obtained during its processing [28,34,35]. According to the available literature, the phenolic fraction of sugar beet roots and pulp is primarily represented by hydroxybenzoic acid derivatives, especially gallic, 4-hydroxybenzoic, and vanillic acids. Hydroxycinnamic acids, such as ferulic and p-coumaric acids, are also present, although usually at lower concentrations.

These characteristics indicate that ESBP may serve as a fiber-rich functional ingredient with additional bioactive properties in gluten-free pasta formulations.

The obtained results indicate that, in addition to their high dietary fiber content, ESBP also represent a source of phenolic compounds and exhibit measurable antioxidant potential. This may justify their use as a functional ingredient in the development of food products with an improved bioactive profile and enhanced antioxidant capacity. According to Baryga et al. [28], sugar beet pulp is characterized by a high content of minerals, with an ash content of approximately 4.16%, as well as considerable amounts of protein (10.11%), fat (6.90%), and dietary fibre (67.60%). In addition, sugar beet pulp contains free sugars, predominantly fructose (1.12 g/100 g) and glucose (0.84 g/100 g). It also contains bioactive compounds with potential health-promoting properties, including polyphenols (366.00–520.02 mg CE/100 g), mainly flavonoids (304.00–370.00 mg RE/100 g) and phenolic acids (up to 250.67 mg FAE/100 g).

Considering that the efficiency of bioactive compound extraction from sugar beet pulp is determined, among other factors, by the type of solvent used [36,37,38], and taking into account the impact of the extrusion process, the content of bioactive compounds determined in the present study was higher than the values reported by Baryga et al. [28], who analyzed raw sugar beet pulp. The total phenolic/polyphenol content, determined both with and without the use of the Folin–Ciocalteu reagent, as well as the content of phenolic acids, was 34%, 22%, and 38% higher, respectively, in the present study than in the work of Baryga et al. [28], namely approximately 1.13 mg CE/g DM for the Folin–Ciocalteu assay, 0.48 mg CE/g DM for the non-Folin method, and 0.13 mg FAE/g DM for phenolic acids, despite the use of a similar extraction method for phenolic compounds. The observed trend may be explained by the effect of the extrusion process, which promotes the release of polyphenolic compounds bound within the plant matrix structure, including fiber complexes [39]. As a result of high temperature, pressure, and shear forces, polyphenols may be partially converted from bound or conjugated forms into free forms, thereby increasing their extractability and analytical availability [40]. Therefore, it can be concluded that the extrusion of sugar beet pulp has a beneficial effect not only on reducing the characteristic taste and aroma of this by-product but also on increasing the efficiency of polyphenol extraction. Consequently, extruded sugar beet pulp may be regarded as a valuable source of dietary fiber and bioactive compounds with potential applications in the development of functional foods.

### 2.2. Bioactive Compound Content and Antioxidant Activity of Conventional and High-Protein Gluten-Free Pasta

#### 2.2.1. Total Polyphenol Content

Polyphenols are among the most important bioactive compounds present in plant-derived raw materials and food products. The total phenolic/polyphenol content in the analyzed gluten-free pasta samples was determined using two independent methods: with the use of the Folin–Ciocalteu (F.C.) reagent and without its use. The F.C. assay measures the overall reducing capacity of the extract and may therefore respond to both mono- and polyphenolic compounds, as well as to non-phenolic reducing substances. In contrast, the method without the F.C. reagent primarily reflects compounds exhibiting characteristic spectrophotometric absorption, although other UV-absorbing matrix constituents may also contribute to the measured signal [41]. The results are presented in Table 2.

In the analyses performed using the Folin–Ciocalteu reagent, an initial decrease in phenolic content was observed in the conventional pasta samples. The addition of 5% and 10% of the enriching ingredient reduced the measured values by approximately 16% and 40%, respectively, compared with the control sample. In contrast, pasta containing 15% of the additive exhibited a phenolic content approximately 72% higher than that of the control. Much greater changes were observed in the high-protein pasta samples. The incorporation of 5% additive resulted in an approximately 80% reduction in phenolic content relative to the control, whereas the 10% addition led to a decrease of about 37%. Only the application of 15% additive produced a phenolic content nearly 47% higher than that of the control sample.

A particularly pronounced difference was observed between the two control samples, with the high-protein control (K_HP) showing a substantially higher value in the F.C. assay than the conventional control (K_N). This difference may be partly associated with the presence of pea protein isolate in the high-protein formulation. Pea protein isolate may contain residual phenolic compounds originating from the raw material; however, its contribution to the F.C. response is unlikely to be attributable exclusively to these compounds. The Folin–Ciocalteu reagent measures the overall reducing capacity of the extract and may also react with reducing amino acids, peptides, protein-derived compounds, and other non-phenolic reducing substances present in the protein isolate. Moreover, compounds formed during processing, including Maillard reaction products, may further increase the measured response. Therefore, the unexpectedly high value recorded for K_HP should be interpreted as an apparent increase in total phenolic content rather than unequivocal evidence of a proportionally higher concentration of phenolic compounds. The fact that the difference between the control samples was most pronounced in the F.C. assay further suggests that matrix-related interference may have contributed to the observed result.

Despite differences in the magnitude of changes, the highest phenolic content determined by the F.C. method was observed at the 15% addition level in both pasta types. This phenomenon may result from interactions between phenols and components of the product matrix, as well as from the partial saturation of binding sites, which increases the proportion of compounds available for determination [42,43]. The sharp decrease in measurable phenolic content at lower ESBP levels, particularly evident in the high-protein system, may indicate strong interactions between proteins, particularly pea protein isolate, and phenolic compounds, leading to their partial “masking” during extraction and analysis [43]. Such interactions may result in the formation of complexes with lower solubility or reactivity in standard analytical assays, thereby reducing the analytically detectable fraction of phenolic compounds [43]. At the 15% ESBP addition level, the higher amount of phenolic compounds introduced with the additive may have exceeded, at least partly, the binding capacity of the matrix, resulting in a more evident enrichment effect. This indicates a non-linear relationship between the amount of introduced raw material and its measurable bioactive potential [44]. In addition, because the F.C. reagent is not specific exclusively to phenolic compounds, changes in the concentrations of other reducing constituents of the pasta matrix may also have contributed to the observed differences.

For the analyses performed without the use of the Folin–Ciocalteu reagent, the addition of 5% of the tested ingredient to conventional pasta did not significantly affect the polyphenol content compared with the control sample. Increasing the additive level to 10% and 15% resulted in a slight increase in polyphenol content of approximately 10% and 12%, respectively, relative to the control. In high-protein pasta, the method without F.C. showed a systematic increase in polyphenol content as the additive level increased up to 10%. A further increase to 15% did not result in additional improvement, and the obtained value was approximately 23% higher than that of the control sample.

In summary, the lower values obtained with the non-F.C. method likely result from differences in analytical principles and its lower sensitivity to non-phenolic reducing compounds, such as amino acids, peptides, reducing sugars, and Maillard reaction products, which may contribute to the F.C. response [41,45,46]. The two methods should therefore be considered complementary, although the non-F.C. approach may also detect other UV-absorbing matrix components. In the high-protein pasta, 10% and 15% ESBP significantly increased the measured phenolic content compared with the control, with no difference between these levels, suggesting a plateau at 10% ESBP.

These results indicate that the pea-protein-rich matrix affected the extraction and analytical detectability of phenolic compounds. Extrusion may also have increased their availability by disrupting bonds between phenolics and cell-wall components, thereby releasing more extractable forms [28].

The study by Conti et al. [47] showed that the enrichment of pasta with plant-based additives, such as carrot powder and olive leaves, increased the total polyphenol content in the product by 57% for carrot and up to 260% for olive leaves. Bavaro et al. [48] evaluated the effect of artichoke addition on polyphenol content in pasta. The obtained results showed a strong enrichment effect. Similar results were reported by Krawęcka [49], who fortified pasta with black cumin pomace and demonstrated a statistically significant increase in polyphenol content already at the 5% addition level, from 0.02 to 1.12 mg GAE/g DM.

In the study by Gumul et al. [50], polyphenol content increased after the incorporation of both lyophilized red potato pulp and lyophilized cherry pomace into pasta, compared with the control group. A higher polyphenol content was observed in pasta with cherry pomace than in pasta with lyophilized red potato pulp. This resulted from the significantly higher polyphenol content in lyophilized cherry pomace than in lyophilized red potato pulp. It should be emphasized that a 30% addition of lyophilized red potato pulp increased the polyphenol content fourfold compared with the control pasta. Similarly, the same level of cherry pomace addition increased the polyphenol content in pasta fivefold compared with the control [50]. After pasta enrichment with fiber from orange by-products, Crizel et al. [51] observed an increase in polyphenol content ranging from 23% to 43% compared with the control group. In contrast, in the study by Baigts-Allende et al. [52], concerning the use of a *Hibiscus sabdariffa* by-product in pasta, the observed increase in polyphenol content was more than tenfold.

#### 2.2.2. Content of Selected Phenolic Compound Fractions

The content of phenolic acids in conventional pasta changed with increasing ESBP addition (Table 3). The addition of 5% ESBP reduced their content by approximately 20% compared with the control, whereas a 10% addition resulted in an increase of approximately 24%. The highest value was recorded for sample N_15%, in which the phenolic acid content was more than three times higher than that of the control sample. In high-protein pasta, the addition of 5% ESBP reduced the phenolic acid content by approximately 95% relative to the control. Increasing the additive level to 10% resulted in a partial recovery of phenolic acid content; however, the value remained approximately 50% lower than that of the control. Only the 15% addition level allowed a phenolic acid content comparable to that of the control sample to be achieved. No statistically significant differences in flavonol content were observed among the conventional pasta samples. In contrast, the addition of ESBP to high-protein pasta resulted in a clear reduction in flavonol content. The application of 15% ESBP partially mitigated this effect; however, the flavonol content remained approximately 24% lower than that of the control sample. Anthocyanins were not detected in any of the analyzed samples. Consequently, no significant differences were observed among the tested variants. The flavonoid content in conventional pasta increased with increasing ESBP addition. In samples N_10% and N_15%, flavonoid levels were approximately 22% and 33% higher, respectively, than in the control. In high-protein pasta, a 5% ESBP addition increased flavonoid content by approximately 22% relative to the control. Further increases in the additive level did not significantly affect flavonoid content. The limited differences in flavonol and flavonoid contents may be attributed to their relatively small contribution to the overall phenolic profile, analytical variability, and reduced extractability resulting from processing and interactions with proteins or polysaccharide [28,43]. This effect may be particularly relevant in the high-protein matrix because of the formation of complexes with pea proteins. In addition, oxygen exposure during thermal processing, particularly during pasta cooking, may contribute to the degradation of these phenolic fractions [53].

The antioxidant activity determined using the DPPH and ABTS assays (Table 4) reflects the combined effects of numerous antioxidant compounds and cannot be attributed solely to flavonols or flavonoids. Therefore, changes in phenolic acid contents and interactions among individual antioxidants may explain similar trends in antioxidant activity despite the absence of significant differences in some phenolic fractions. Other compounds formed during pasta production, including Maillard reaction products, may also contribute to the measured antioxidant activity. Furthermore, changes in antioxidant capacity may result from the conversion of phenolic compounds from less active forms into more active forms, such as aglycones, as well as from the presence of other bioactive constituents [41,54].

The increase in phenolic compound content observed in the enriched pasta samples is consistent with previous findings. Gumul et al. [55] demonstrated that the incorporation of plant-based ingredients into gluten-free formulations can significantly enhance the phenolic compound content of the final product. In gluten-free bread containing 5% dried apple pomace, the authors observed a 2.5-fold increase in total polyphenol content and an 8-fold increase in flavonoid content compared with the control sample. These findings indicate the effectiveness of plant-derived materials in enhancing phenolic fractions in gluten-free foods. Similar observations were reported by Benchettah et al. [7], who enriched gluten-free rice pasta with almond and tomato by-products and obtained a product with a substantially improved antioxidant profile. Likewise, Lawrence et al. [44] demonstrated that the addition of powdered mango leaves to a gluten-free matrix not only markedly increased the content of polyphenols, including the valuable compound mangiferin, but also imparted antidiabetic properties through the inhibition of α-glucosidase activity. The use of spirulina biomass [19] enabled the production of pasta with enhanced nutritional density, rich in phycocyanin and β-carotene, which significantly increased the antioxidant potential of the final product. Relating these observations to the present findings, it can be assumed that the differences observed among the ESBP-enriched variants result from both the amount of phenolic compounds introduced with the additive and their availability within the product matrix. This may affect the levels of individual phenolic fractions determined in the samples. According to Obuchowski et al. [56], pasta enriched with mulberry extracts exhibited a significant increase in phenolic acid content, in some cases reaching values twice as high as those of the control sample. Further studies on the use of bergamot pomace [57] and flaxseed press cake [18] confirmed that an appropriate additive level, such as 10–15%, can ensure high flavonoid stability, allowing these compounds to remain active even after cooking. These findings indicate that the effectiveness of pasta fortification with phenolic compounds is strongly dependent on the characteristics of the plant-based additive used. Natural ingredients rich in polyphenols, such as mulberry, mango leaves, and algae, may substantially increase the concentration of bioactive compounds and the antioxidant potential of pasta products [19,44,56,58].

#### 2.2.3. Antioxidant Activity Determined by DPPH and ABTS Assays

The antioxidant activity determined by the DPPH assay in conventional pasta gradually decreased with increasing levels of ESBP addition. In samples N_5%, N_10%, and N_15%, the values were approximately 10%, 9%, and 15% lower, respectively, than in the control sample. In high-protein pasta, the changes were much less pronounced. Samples HP_5% and HP_10% did not differ significantly from the control, whereas the application of 15% ESBP resulted in an approximately 4% increase in antioxidant activity relative to the control sample. Different trends were observed for the ABTS assay. In conventional pasta, antioxidant activity increased with increasing ESBP addition, reaching the highest value in sample N_15%. The N_5%, N_10%, and N_15% variants exhibited approximately 2-, 6-, and 8-fold higher antioxidant activity, respectively, than the control sample. In high-protein pasta, the highest antioxidant activity was recorded in the control sample. The addition of 5% and 10% ESBP reduced antioxidant activity by approximately 43% and 48%, respectively, whereas the 15% addition partially limited this decrease. Despite this improvement compared with the other enriched variants, the antioxidant activity of sample HP_15% remained approximately 35% lower than that of the control.

The lower antioxidant activity determined by the DPPH and ABTS assays in the final pasta products may be associated with limited retention and/or extractability of antioxidant compounds within the pasta matrix. Since only cooked samples were analyzed and no untreated counterpart was included, the specific contribution of thermal processing cannot be distinguished from matrix-related effects. To partially compensate for these losses, increasing the ESBP content may be justified. However, the effectiveness of such an approach should be considered together with the effect of the additive on product structure stability and the leaching of components during cooking. The discrepancy between the trends observed with the DPPH and ABTS methods has been reported in the literature and results from differences in the properties of the radicals used and the reaction conditions. Consequently, these assays may reflect the activity of different groups of antioxidants [59]. In the study by Moo-Huchin et al. [60], the incorporation of *Enterolobium cyclocarpum* flour into wheat pasta resulted in a clear increase in antioxidant capacity measured by the ABTS assay. No antioxidant activity was detected in the control pasta, whereas enriched samples exhibited values ranging from 16 to 62 μM Trolox equivalents/100 g, increasing with the level of additive incorporation.

Koli et al. [61] demonstrated that enrichment of pasta with spirulina biomass significantly increased its antioxidant potential, as determined by the DPPH and FRAP assays. DPPH radical scavenging activity in the enriched pasta samples ranged from approximately 43% to 51%, whereas ferric reducing antioxidant power measured by the FRAP assay ranged from approximately 2.5 to 5.1 μmol TE g^−1^. These values were clearly higher than those observed in the control sample and increased with increasing levels of spirulina addition.

According to the study conducted on pasta enriched with black cumin pomace [49], radical scavenging activity increased with the level of additive incorporation. When black cumin pomace was added at a level of 25%, antioxidant activity increased by approximately 70%. Significant improvements were already observed at a 15% addition level.

The study by Bavaro et al. [48] demonstrated that the enrichment of wheat pasta with artichoke extract led to a substantial increase in antioxidant activity. These findings confirm that the use of natural raw materials rich in polyphenols can significantly improve the bioactive properties of pasta products, both in terms of antioxidant capacity and their potential protective effects against oxidative stress.

#### 2.2.4. HPLC Profile of Polyphenols in ESBP-Enriched Gluten-Free Pasta

In addition to the spectrophotometric analyses of polyphenol content, the qualitative and quantitative profiles of polyphenolic compounds were determined using liquid chromatography. HPLC analysis confirmed the presence of hydroxybenzoic and hydroxycinnamic acids in both extruded sugar beet pulp (ESBP) and gluten-free products enriched with this additive. The predominant compounds were gallic, 4-hydroxybenzoic, ferulic, vanillic, and p-coumaric acids (Table 5).

Analysis of the above-mentioned phenolic acids in pasta containing 5–15% ESBP showed that the addition of 5–10% ESBP resulted only in minor, statistically non-significant changes in their contents. A significant increase was observed only at the 15% addition level, particularly for ferulic, gallic, vanillic, and 4-hydroxybenzoic acids, whose contents increased by 6%, 26%, 37%, and 43%, respectively, compared with the control (Table 5). A similar trend was observed in the high-protein pasta, in which the incorporation of 15% ESBP produced the greatest increases in ferulic and vanillic acid contents (Table 5). In the case of gallic acid, even a 5% ESBP addition resulted in an increase of approximately 48% compared with the corresponding control pasta. Moreover, regardless of the ESBP addition level, the gallic acid content in the enriched high-protein pasta was, on average, approximately 50% higher than that in the control sample. Generally, the HPLC results were consistent with those obtained using the spectrophotometric methods, as both analytical approaches showed that the addition of 15% ESBP increased the contents of phenolic compounds and phenolic acids in both types of pasta compared with their respective controls (Table 2, Table 3 and Table 5).

The relatively low concentrations of the individual phenolic acids may appear disproportionate to the high total phenolic content and antioxidant activity measured using the DPPH and ABTS assays. This discrepancy can be explained by the different analytical principles of the methods applied. High-performance liquid chromatography with diode-array detection (HPLC-DAD) enables the determination of selected free phenolic compounds present in the extract, whereas the Folin–Ciocalteu method and antioxidant activity assays reflect the overall reducing capacity of the sample, resulting from the combined activity of all compounds with antioxidant properties [62].

It should also be emphasized that sugar beet and sugar beet pulp obtained during its processing differ markedly from many other plant materials, such as wheat or rye bran, in terms of their phenolic composition [28,34,35,63]. Sugar beet pulp has a considerably less diverse phenolic profile, and its total content of free phenolic acids is naturally low. Most phenolic compounds remain bound to the dietary fibre fraction, including cellulose, hemicelluloses, lignin, and pectic substances. Numerous studies have shown that, in matrices such as sugar beet pulp, more than 80–90% of the total phenolic pool may occur in bound forms that are not detected by direct HPLC analysis following hydroalcoholic extraction [64,65].

The profile of phenolic compounds identified in the present study is consistent with the literature data concerning sugar beet and its processing products [28,34,35]. Previous studies have confirmed that sugar beet roots and sugar beet pulp contain mainly hydroxybenzoic acids, including gallic, 4-hydroxybenzoic, and vanillic acids, together with smaller quantities of hydroxycinnamic acids, particularly ferulic and *p*-coumaric acids. At the same time, the total content of free phenolic acids in this raw material is relatively low compared with other plant materials rich in phenolic compounds, such as cereal bran and berries [66,67,68,69].

In this context, both the extraction procedure used for phenolic compound determination [70] and the effect of extrusion applied for the microbiological stabilization of sugar beet pulp should be considered. This thermo-mechanical treatment induces several opposing processes simultaneously, including the partial degradation of thermolabile phenolic compounds, the release of phenolics bound to the fibre matrix, their oxidation and polymerization, and the formation of new compounds with antioxidant properties. Of particular importance is the formation of Maillard reaction products, especially melanoidins, which exhibit considerable antioxidant activity and may substantially increase the overall antioxidant potential of ESBP and ESBP-enriched products. Therefore, the high antioxidant activity of the analyzed samples cannot be attributed solely to the free phenolic acids determined by HPLC (or TPC by spectrophotometric methods) but should instead be regarded as the combined effect of several groups of bioactive compounds present in the samples.

It is also noteworthy that, despite the relatively low absolute concentrations of the individual phenolic acids, their qualitative profile was maintained across all analyzed samples, while the incorporation of 15% ESBP increased the contents of most of the identified phenolic compounds. These findings indicate that the phenolic profile remained relatively stable during processing and confirm that ESBP can serve as a source of natural antioxidants in gluten-free products.

#### 2.2.5. Physical Properties of Gluten-Free Pasta Containing ESBP: Texture and Color

Product texture, including attributes such as firmness, hardness, chewiness, and adhesiveness, plays an important role in shaping consumer perception by influencing sensory attributes and overall product acceptability [49]. Instrumental texture analysis (Table 6) showed that the N_10% sample exhibited significantly higher firmness, expressed as the maximum cutting force, than the remaining conventional pasta formulations. In contrast, no significant differences in firmness were observed among the high-protein pasta samples. The increased firmness of N_10% may indicate that the 10% ESBP level provided a favorable balance between starch, dietary fibre, and water within the conventional pasta matrix. At this concentration, non-starch polysaccharides present in ESBP, particularly pectins and arabinans, may have enhanced water retention and promoted stronger interactions with gelatinized starch, resulting in a more compact and resistant structure. The lower firmness observed at the 5% addition level may reflect an insufficient amount of fibre to produce a measurable reinforcing effect, whereas at 15% ESBP, the larger proportion of fibre may have competed with starch for water and partially disrupted the continuity of the starch-based matrix. Therefore, the effect of ESBP on firmness appears to be non-linear, with the 10% addition level providing the most favorable structural reinforcement in conventional pasta. At the same time, an increasing trend in cutting work was observed. This parameter reflects the total energy required for the blade to pass through the product. The effect was particularly evident in the high-protein variant with the highest level of additive incorporation (sample HP_15%), which may indicate increased cohesiveness of the pasta matrix. The improved structural stability may be attributed to the presence of non-starch polysaccharides in ESBP, particularly arabinans and pectins [28]. Unlike citrus pectins, sugar beet pectins are characterized by a high degree of acetylation and the presence of protein residues, which impart specific emulsifying and stabilizing properties [71]. These components, together with the loosened fiber structure resulting from extrusion, may enhance water-binding capacity and support the formation of a cohesive starch-based matrix, thereby reducing brittleness in gluten-free pasta systems. The stability of extruded pasta depends on the degree of interaction between ingredients and processing parameters, which in turn affects the final physicochemical characteristics and quality of the finished product [72,73].

The effect of food industry by-products on pasta texture varies and depends on the chemical characteristics of the additive. Tolve et al. [74] demonstrated that the addition of grape pomace affected the mechanical properties of pasta, resulting in increased firmness and adhesiveness. The authors attributed these changes to the interaction of the dietary fiber fraction with the matrix structure and competition for water during cooking. Microstructural observations revealed a less organized protein network in the enriched samples, which explained the observed textural modifications. Bianchi et al. [75] highlighted the potential of agri-food by-products as natural texture enhancers that promote increased firmness and structural stability after cooking. A similar effect was reported by Wójtowicz et al. [76] in gluten-free snack products, where the addition of elderberry, chokeberry, and strawberry materials resulted in a substantial increase in firmness and bulk density, indicating improved structure and a higher content of solid components. However, these relationships are not always linear. Benchettah et al. [7] enriched rice pasta with almond by-products and obtained firmness values comparable to those of the control sample, similarly to the results observed in the present study for ESBP. In contrast, the addition of tomato pomace reduced pasta firmness. A similar decrease in hardness was reported for pasta fortified with fish powder [19], which was attributed to starch dilution and the formation of a softer protein matrix. Interesting observations were also reported by Lawrence et al. [44], who found that despite differences in starch retrogradation potential among various mango leaf cultivars, the instrumental cutting force of spaghetti remained at a similar level. This suggests that the presence of plant fibers may mask differences resulting from the properties of starch itself. In pasta enriched with carrot powder [77], hardness increased in fresh samples but decreased in dried products, highlighting the importance of technological processing in shaping the final texture.

**Figure 3:**
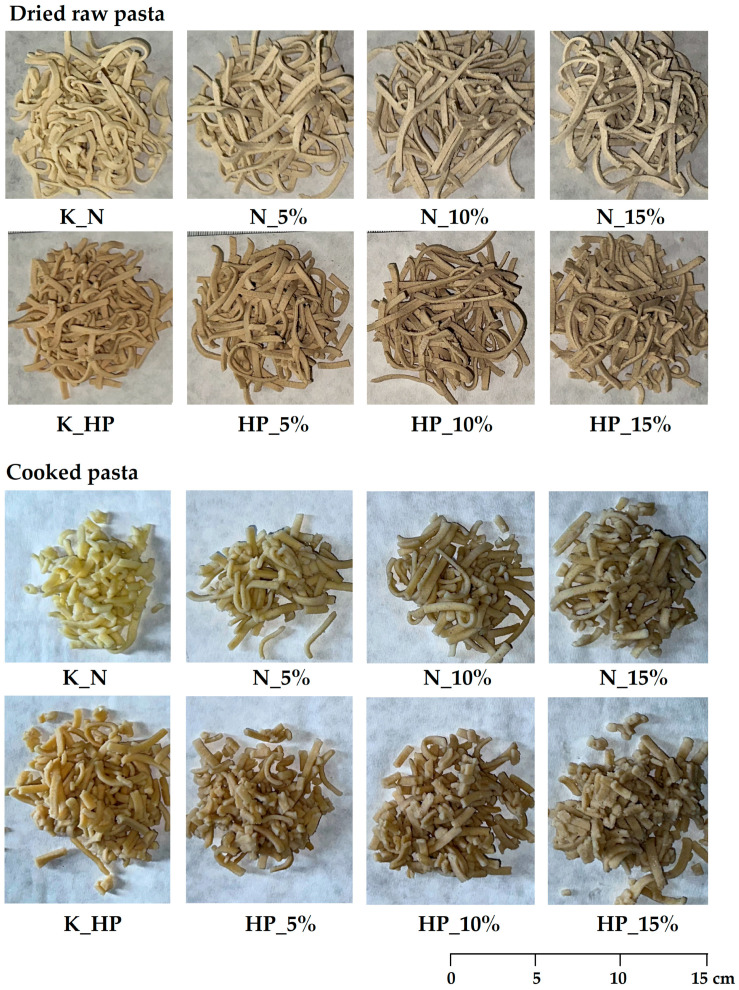
Photographs of dried raw and cooked conventional and high-protein gluten-free pasta samples containing different levels of ESBP.

During the preparation of conventional and high-protein pasta samples enriched with sugar beet pulp, the color of both raw pasta and cooked pasta after the drying process was evaluated (Figure 3, Table 7). The color of the fresh uncooked samples was measured to determine the direct effect of sugar beet pulp incorporation before cooking and to enable comparison with the color of the processed product.

The addition of ESBP significantly affected the color of both conventional and high-protein pasta in their raw and cooked forms. In raw conventional pasta, increasing the ESBP level resulted in decreases in L*, a*, and b* values, indicating product darkening and a reduction in the red and yellow color components. After cooking, the decrease in lightness became even more pronounced, whereas the a* parameter increased relative to the control, particularly at higher additive levels. In high-protein pasta, ESBP addition also reduced L*, a*, and b* values. However, the decrease in lightness in the raw samples was less pronounced than in conventional pasta. After cooking, high-protein pasta samples enriched with ESBP exhibited lower lightness values than the control, although differences among the ESBP levels were relatively small. The greatest changes were observed for the a* and b* parameters, particularly at the 15% ESBP level. The color changes observed in ESBP-enriched pasta cannot be attributed solely to the presence of natural pigments in the pulp. Darkening may also result from Maillard reactions and the oxidation of phenolic compounds occurring during extrusion and subsequent hydrothermal treatment during cooking. Since ESBP contains sugars (18% d.m.; Table 1), it may provide substrates involved in non-enzymatic browning reactions under thermal processing conditions. Overall, compared with conventional pasta, the high-protein variants generally exhibited lower lightness and a greater contribution of the red color component, especially in the raw form. Cooking reduced the lightness of both pasta types and altered the contribution of individual chromatic parameters. The ΔE_00_ values confirmed visible color differences relative to the control samples. In raw conventional pasta, the addition of 5% ESBP addition resulted in a moderate color difference that was clearly perceptible to the observer, whereas 10% and 15% ESBP additions caused large and easily noticeable differences. After cooking, all ESBP levels produced substantial color differences compared with the control sample. In raw high-protein pasta, the addition of 5% ESBP addition caused a small but noticeable color difference, whereas 10% and 15% ESBP additions resulted in moderate color differences. After cooking, a 5% ESBP addition still produced a moderate color difference, while 10% and 15% additions exceeded the threshold for a large and easily perceptible color change.

According to Obuchowski et al. [56], the addition of mulberry and nettle extracts significantly modified the color profile of cereal products by decreasing L* values and increasing the red color component (a*), which was attributed to the presence of natural pigments. Similarly, Florença et al. [77] demonstrated that carrot-enriched pasta develops an intense orange coloration, which is more pronounced in wheat-based products than in buckwheat-based products. Tolve et al. [74], investigating semolina pasta enriched with grape pomace, reported decreases in L* and b* values and an increase in a*, indicating product darkening and a shift toward red coloration. Comparable effects were described by Benchettah et al. [7] for rice pasta enriched with tomato pomace and by Gattuso et al. [57] for pasta containing bergamot pomace. In these studies, color changes were associated with Maillard reactions, pigment degradation, and oxidation processes. Color modifications have also been observed with other functional ingredients. Hussein et al. [19] showed that spirulina imparts a green color to quinoa pasta while simultaneously reducing its lightness. Emir Çoban et al. [20] reported slight darkening of buckwheat pasta enriched with powdered trout meat. Lawrence et al. [44] highlighted the importance of raw material cultivar, demonstrating differences in color stability among spaghetti samples enriched with mango leaves. Moo-Huchin et al. [60] showed that increasing the proportion of Guanacaste seed flour significantly modified color saturation, which was attributed to the presence of phenolic compounds. Taken together, these findings indicate that the observed darkening of samples and the modification of chromatic parameters with increasing additive levels follow the typical pattern of changes induced by ingredients rich in dietary fiber and natural pigments.

#### 2.2.6. Cooking Properties of Gluten-Free Pasta Containing ESBP

The optimum cooking time (OCT), defined as the time required for the disappearance of the opaque white core, was determined separately for each formulation. It ranged from 6 min 30 s to 7 min 30 s for the conventional pasta samples and from 6 min 30 s to 8 min for the high-protein pasta samples.

The incorporation of sugar beet pulp into the gluten-free pasta formulation significantly affected the measured cooking properties (Table 8). Changes in the swelling index (SI), water absorption (WA), and weight increase after cooking (WIC) reflected differences in hydration and swelling during cooking, whereas cooking loss (CL) provided an indirect measure of the ability of the pasta matrix to retain soluble components.

The addition of ESBP affected the cooking properties of both pasta types; however, the magnitude of the changes depended on the matrix composition. In conventional pasta, a 5% ESBP addition did not cause significant changes in SI, WA, or WPM compared with the control sample. At 10% and 15% addition levels, these parameters increased markedly. Compared with the control, SI increased by approximately 15% and 20%, WA by approximately 14% and 19%, and WPM by approximately 9% and 12%, respectively. These results indicate that higher levels of ESBP improved the water-binding capacity of conventional pasta and increased its weight increase parameter during cooking. Cooking losses also increased at higher additive levels, reaching a value approximately 45% higher than the control at 15% ESBP. However, due to variability in the results, the differences relative to the control were not statistically conclusive.

In high-protein pasta, the increases in SI, WA, and WPM were more gradual. At 5% and 10% ESBP addition levels, SI and WPM values were higher than those of the control, but the differences were not statistically significant. A more pronounced effect was observed only at the 15% ESBP level, where SI increased by approximately 29%, WA by approximately 23%, and WPM by approximately 19% compared with the control. At the same time, this sample exhibited the highest cooking losses, which were nearly 2.9 times greater than those of the control. This indicates reduced structural stability of the high-protein pasta at the highest additive level. The simultaneous increase in swelling index, water absorption, and cooking losses at the highest ESBP level may be explained by the specific structural properties of sugar beet pulp fiber. The high content of non-starch polysaccharides, particularly pectins and arabinan-rich fractions, together with the fiber structure loosened by extrusion, may increase the number of available hydrophilic sites and enhance water uptake during cooking [71]. However, at the 15% substitution level, excessive fiber incorporation may physically disrupt the continuity of the starch-based matrix. This interference can limit the formation of a compact and protective network, thereby facilitating the leaching of amylose and other soluble solids into the cooking water.

When the two pasta types were compared, the high-protein variants generally showed lower water absorption and lower weight increase than their corresponding conventional counterparts. In the high-protein control sample, SI was approximately 9% lower, WA approximately 8% lower, and WIC approximately 15% lower than in conventional pasta. A similar trend was observed in the ESBP-enriched samples, particularly at the 10% addition level, where high-protein pasta exhibited approximately 13% lower SI, 12% lower WA, and 17% lower WIC than conventional pasta. At the same time, high-protein pasta was more susceptible to cooking losses, especially at the 15% ESBP level, where CL was almost twice as high as in the corresponding conventional pasta.

Previous studies indicate that gluten-free pasta often exhibits high water absorption and cooking losses because the absence of gluten limits the integrity of the gelatinized starch matrix [77,78]. Cooking losses of approximately 5–14% have been reported for buckwheat and rice pasta, as well as for pasta produced from dent maize varieties, particularly when fibre-rich plant by-products were incorporated [7,77,79]. In contrast, protein-rich additives may improve matrix stability and reduce solids loss during cooking [20]. The swelling index reported for corn pasta was similar to that observed in the present study, whereas the higher water absorption of ESBP-enriched pasta may be related to the strong water-binding capacity of the added dietary fibre [62]. Comparable increases in weight gain have also been observed in gluten-free pasta containing spirulina or extruded rice–pea flour blends [19,80].

#### 2.2.7. Effect of ESBP Addition on Consumer Preferences Toward Conventional and High-Protein Gluten-Free Pasta

Consumer evaluation showed that ESBP addition affected the acceptance of the pasta samples, although the direction of the changes depended on the type of matrix and the sensory attribute evaluated (Table 9). In conventional pasta, ESBP addition did not reduce appearance scores; however, it decreased color and taste ratings. The greatest reduction in taste score compared with the control, approximately 37%, was observed at the 15% ESBP level. In contrast, firmness ratings were higher in the enriched samples, particularly at 5% and 15% ESBP, where the increase was approximately 28%.

In high-protein pasta, the sample containing 10% ESBP received the highest appearance score, which was approximately 22% higher than that of the control. However, ESBP addition reduced ratings for color, aroma, and taste. The most unfavorable effect was observed at the 15% addition level. In this sample, overall acceptability was approximately 35% lower than that of the control.

When comparing the two pasta types, very similar sensory scores were obtained at the 5% ESBP level. At the 10% addition level, high-protein pasta received higher ratings for appearance, firmness, and overall acceptability. However, at the 15% ESBP level, its acceptance was lower than that of the corresponding conventional pasta.

Similar relationships were reported by Gattuso et al. [57], who found that bergamot pomace additions above 2.5% intensified bitter notes and negatively affected product appearance due to discoloration. Likewise, in quinoa pasta enriched with spirulina [19] and in sorghum pasta [81], higher levels of additives reduced taste and aroma scores because of excessively intense plant-derived flavors and the bitterness of polyphenols. In contrast, Bongianino et al. [79] confirmed that high firmness is a key factor influencing consumer preference for gluten-free pasta, which is consistent with the favorable perception of firmness observed in the ESBP-enriched variants in the present study. It should be noted, however, that the application of appropriate technological approaches, such as vinegar deodorization in trout-enriched pasta [20] or the addition of carrot powder [82], can maintain high product acceptability even at enrichment levels of 10–15%.

## 3. Materials and Methods

### 3.1. Materials

The research material consisted of sugar beet pulp and gluten-free pasta samples. The sugar beet pulp was obtained from Lutkala Sp. z o.o. (Nasielsk, Poland) and subjected to extrusion using a co-rotating twin-screw extruder manufactured by Fudex (model 2FS60, Cavriago, Italy). The extruded material was subsequently milled and sieved to obtain a particle size fraction of 300 μm. The extrusion feedstock consisted exclusively of sugar beet pulp with a moisture content of approximately 20%, without the addition of starch-based ingredients. The raw material was fed into a twin-screw extruder equipped with an extrusion die. The barrel was heated in consecutive zones to temperatures ranging from 105 °C to 180 °C, while the screw speed was maintained at 800 rpm. The detailed temperature settings were not disclosed, as they constitute proprietary know-how associated with Polish patent application P.429586, entitled “Use of extruded waste fruit and/or vegetables as thickeners” [83].

Gluten-free pasta was prepared using a mixture of rice flour (Naturalnie Zdrowe Sp. z o.o., Wiązowna, Poland), corn flour (Naturalnie Zdrowe Sp. z o.o., Poland), potato flour (PPZ Trzemeszno Sp. z o.o., Trzemeszno, Poland), and tapioca flour (Naturalnie Zdrowe Sp. z o.o., Poland). The formulations also included pea protein isolate (Pięć Przemian—Simpatiko Sp. z o.o., Dąbrowa, Poland), extruded sugar beet pulp (Lutkala Sp. z o.o., Nasielsk, Poland), Kujawski rapeseed oil (Bunge Polska Sp. z o.o., Kruszwica, Poland), guar gum (Naturalnie Zdrowe Sp. z o.o., Poland), eggs, salt, and water. To evaluate the effect of extruded sugar beet pulp on the cooking and bioactive value of conventional and high-protein pasta, part of the base material was replaced with 5%, 10%, and 15% ESBP. A control sample was also prepared. The ingredients were mixed according to the formulation presented below (Table 10) using a planetary mixer (Kenwood Ltd., Havant, UK) for approximately 20 min at low speed. The amount of water added was determined empirically (organoleptically) during the mixing process to achieve an optimal dough consistency suitable for rolling and cutting, as the water absorption capacity varied depending on the substitution level of ESBP and the type of protein matrix. After this period, the dough was removed, rolled to a thickness of 3 mm, and cut using a manual pasta machine to obtain fettuccine-type pasta. The resulting pasta was dried in a drying chamber for 8 h at 40 °C until a final moisture content of approximately 12.5% was reached.

To determine the content of bioactive compounds, the pasta samples were cooked and subsequently subjected to freeze-drying (Labconco, Kansas City, MO, USA) and milling according to the method described by Hirawan et al. Approximately 100 g of dry pasta was cooked in 2000 cm^3^ of boiling distilled water containing 14 g of NaCl for standardized cooking time of 6 min 30 s. This reference cooking time was applied to all pasta formulations to ensure comparable conditions during the determination of cooking properties. It was selected on the basis of preliminary observations of pasta hydration and the disappearance of the visible white, unhydrated core. After cooling, the cooked pasta was frozen at −20 °C and freeze-dried for 24 h using a Labconco FreeZone 6 freeze dryer (Labconco, Kansas City, MO, USA) at −47 °C and a pressure of 0.370 mBar. The freeze-dried samples were subsequently vacuum-sealed in heat-sealed packages stored at room temperature until further analyses.

To facilitate the identification of the analyzed variants, a unified coding system was applied that considered both the type of pasta matrix and the level of fortification with extruded sugar beet pulp (expressed as a percentage of flour weight).

For conventional gluten-free pasta, the following designations were used:

- K_N—control sample (without ESBP addition),
- N_5%, N_10%, and N_15%—conventional gluten-free pasta containing 5%, 10%, and 15% ESBP, respectively.

For high-protein gluten-free pasta, the following codes were applied:

- K_HP (Control High Protein)—control sample,
- HP_5%, HP_10%, and HP_15%—high-protein gluten-free pasta enriched with ESBP at levels of 5%, 10%, and 15%, respectively.

### 3.2. Methods

#### 3.2.1. Determination of Basic Nutrients of ESBP

The content of basic nutrients in the analyzed extruded sugar beet pulp was determined using AOAC methods [84]. Protein (N × 6.25) was measured by the Kjeldahl method [84] using a Kjeltec 2200 extraction unit (Foss, Hillerød, Denmark). Total carbohydrate content was determined by the AOAC method no. 974.06. Fat content was measured by the Soxhlet method (AOAC method no. 953.38) using a Soxtec Avanti 2055 unit (Foss, Denmark). Ash content was determined by AOAC method no. 930.05.

The energy value (1) of the ESBP was calculated according to the guidelines provided in EU Directive 1169/2011 [85,86]. The following caloric values were assigned to macronutrients:

- Available carbohydrates: 4 kcal/g
- Protein: 4 kcal/g
- Fat: 9 kcal/g
- Fiber: 2 kcal/g

The total energy value was computed as

(1) Energy (kcal/100 g)=(carbohydrates×4)+(protein×4)+(fat×9)+(fiber×2)

All of the above measurements were performed in at least two replicates.

#### 3.2.2. Extractions of Polyphenols

For extraction, 0.6 g of the research material was weighed and mixed with 30 cm^3^ of 80% ethanol solution. The process was carried out for 2 h at room temperature (approximately 20 °C), with limited access to light, using a shaking water bath (Memmert WB 22, Memmert GmbH + Co. KG, Schwabach, Germany). After extraction, the samples were centrifuged in a laboratory centrifuge (MPW-350, MPW Med. Instruments, Warsaw, Poland) for 15 min at 4500 rpm (1050× *g*). The obtained supernatant was separated and stored at −20 °C until further analyses.

#### 3.2.3. Determination of Total Phenolic Content

To provide a reliable assessment of the bioactive profile of the enriched gluten-free pasta, the total content of phenolic compounds was determined using two independent spectrophotometric methods. The Folin–Ciocalteu (F.C.) reagent-based method was applied as a widely recognized standard for evaluating the total reducing capacity of extracts. The results obtained using this approach (TPC) were complemented by an analysis performed without the F.C. reagent, as described in Section 3.2.9, at a wavelength of λ = 280 nm. This approach allowed potential interferences from matrix components, such as proteins, free amino acids, or Maillard reaction products, to be minimized, as these compounds may overestimate the results obtained with the classical F.C. method. This is particularly important in the case of the high-protein matrices analyzed in this study.

The total phenolic content was determined spectrophotometrically using the Folin–Ciocalteu reagent, according to the procedure described by Singleton et al. [87]. A sample was taken from 5 cm^3^ of ethanol extract and then diluted with distilled water to a final volume of 50 cm^3^. The Folin–Ciocalteu reagent was prepared by dilution with distilled water at a volume ratio of 1:1.

To 5 cm^3^ of the diluted extract, 0.25 cm^3^ of the prepared Folin–Ciocalteu reagent and 5 cm^3^ of 7% sodium carbonate solution (Na_2_CO_3_) were added. The mixture was thoroughly mixed and then incubated for 30 min under limited light exposure. After incubation, the absorbance of the samples was measured at λ = 760 nm using a spectrophotometer (Helios Gamma, 100–240, Runcorn, UK). An external calibration curve was prepared under the same analytical conditions using catechin standard solutions in the concentration range of 0–100 mg/L. Absorbance (y) was plotted against catechin concentration (x), yielding the calibration equation y = 0.0174x − 0.0001 (R^2^ = 0.998). The phenolic content of the extracts was calculated by interpolation from this calibration curve, taking into account the dilution factor and sample dry matter. No extinction coefficient was used. The results were expressed as milligrams of catechin per gram of dry matter (mg catechin/g DM).

#### 3.2.4. Determination of Flavonoid Content by the Spectrophotometric Method

Flavonoid content was determined spectrophotometrically according to the procedure described by El Hariri et al. [88]. A 0.5 cm^3^ aliquot of ethanol extract was transferred into a test tube, followed by the addition of 1.8 cm^3^ of distilled water and 0.2 cm^3^ of 2-aminoethyl diphenylborinate solution. The obtained mixture was thoroughly homogenized using a Vortex mixer (WF2, Janke & Kunkel, Staufen, Germany).

Absorbance was measured at λ = 404 nm using a Helios Gamma spectrophotometer (100–240, Helios Gamma, Runcorn, UK). A reference sample was prepared in parallel and consisted of 0.5 cm^3^ of 80% ethanol, 1.8 cm^3^ of distilled water, and 0.2 cm^3^ of 2-aminoethyl diphenylborinate solution. An external calibration curve was prepared using rutin standard solutions within the concentration range of 0–100 mg/L. Absorbance (y) was plotted against rutin concentration (x), yielding the equation y = 37.183x + 0.0936 (R^2^ = 0.997). Flavonoid content was calculated by interpolation from the calibration curve, corrected for the dilution factor and sample dry matter. No extinction coefficient was used. The results were expressed as milligrams of rutin per 100 g of dry matter (mg rutin/100 g DM).

#### 3.2.5. Determination of TPC and Selected Phenolic Compound Fractions

The determination of total polyphenol content (TPC), phenolic acids, flavonols, and anthocyanins was carried out according to the method described by Mazza et al. [84], with the modification proposed by Oomah et al. [89]. The analysis was performed spectrophotometrically. A 0.1 cm^3^ aliquot of ethanol extract was transferred into a test tube, followed by the addition of 2.4 cm^3^ of 2% HCl solution in 75% ethanol. The obtained mixture was thoroughly mixed using a Vortex mixer (WF2, Janke & Kunkel, Staufen, Germany).

Absorbance was measured using a Helios Gamma spectrophotometer (100–240, Runcorn, UK) at the following wavelengths:

λ = 280 nm—total polyphenol content (TPC)
λ = 320 nm—phenolic acids
λ = 360 nm—flavonols
λ = 520 nm—anthocyanins

A 2% HCl solution in 75% ethanol with the addition of 80% ethanol was used as the blank sample. For the spectrophotometric determination of UV-absorbing phenolic compounds according to the Mazza–Oomah method [84,89], separate external calibration curves were prepared using standard solutions of catechin, ferulic acid, quercetin, and cyanidin-3-glucoside. Absorbance (y) was plotted against the concentration of the corresponding standard (x). The following calibration equations were obtained: catechin, y = 30.398x − 0.1588 (R^2^ = 0.997); ferulic acid, y = 4.2055x − 0.0148 (R^2^ = 0.995); quercetin, y = 5.0083x − 0.0190 (R^2^ = 0.997); and cyanidin-3-glucoside, y = 13.685x + 0.0092 (R^2^ = 0.997). The concentrations were calculated by interpolation from the respective calibration curves, corrected for the dilution factor and sample dry matter; extinction coefficients were not used. The results were expressed as mg catechin equivalents (CE)/100 g DM, mg ferulic acid equivalents (FAE)/100 g DM, mg quercetin equivalents (QE)/100 g DM, and mg cyanidin-3-glucoside equivalents (C3GE)/100 g DM, respectively [84,89].

#### 3.2.6. Determination of Antioxidant Capacity

##### DPPH Method

The antioxidant activity of the ethanol extracts was determined using the synthetic radical 2,2-diphenyl-1-picrylhydrazyl (DPPH), according to the method described by Brand-Williams et al. [90]. For each determination, 1 cm^3^ of extract was mixed with 4 cm^3^ of DPPH solution prepared by dissolving 0.012 g of DPPH in 100 cm^3^ of ethanol. After thorough mixing, absorbance was measured at λ = 515 nm using a Helios Gamma spectrophotometer (100–240, Runcorn, UK). Trolox (6-hydroxy-2,5,7,8-tetramethylchroman-2-carboxylic acid) was used as the reference standard, and the calibration curve showed a coefficient of determination of R^2^ = 0.98. The Trolox calibration curve used for calculations was y = −14.531x + 0.7184 with a coefficient of determination R^2^ = 0.982. The results were expressed as milligrams of Trolox equivalent antioxidant capacity (TEAC) per gram of dry matter (DM).

##### ABTS Method

Antioxidant activity was determined using the synthetic ABTS•^+^ radical (2,2′-azinobis(3-ethylbenzothiazoline-6-sulfonic acid)) according to the method of Re et al. [91]. Appropriately diluted the ethanol extracts were mixed with the ABTS solution and homogenized using a Vortex mixer (WF2, Janke & Kunkel, Staufen, Germany). Absorbance was measured using a Helios Gamma spectrophotometer (100–240, Runcorn, UK) at λ = 734 nm. A second reading was taken after 6 min at the same wavelength. The antioxidant activity was calculated based on the Trolox calibration curve: y = 4 × 10^6^x + 1.2502 (R^2^ = 0.996). The obtained results were expressed as milligrams of Trolox equivalent antioxidant capacity (TEAC) per gram of dry matter (DM).

#### 3.2.7. Determination of Phenolic Compounds by HPLC-DAD

Phenolic compounds present in sugar beet pomace and in products containing sugar beet pomace were determined using high-performance liquid chromatography with diode-array detection (HPLC-DAD), according to the method described by Ciurlă et al. [92], Oracz et al. [93] and Whelan et al. [94] with modifications adapted to the analyzed matrix.

Prior to chromatographic analysis, the extracts were filtered through 0.22 μm PTFE syringe filters and transferred into HPLC vials. Chromatographic separation was performed using an Ultimate 3000 HPLC system (Dionex, Thermo Fisher Scientific, Germering, Germany) equipped with UV-Vis diode-array detector (DAD). Separation was carried out on an Accucore™ C18 column (150 × 2.1 mm, 2.6 μm particle size; Thermo Fisher Scientific, USA), maintained at 28 °C. The injection volume was 2 μL, while the flow rate was 0.35 mL min^−1^.

The mobile phase consisted of (A) 1% (*v*/*v*) formic acid in water and (B) acetonitrile. Elution was performed using the following gradient program: 0 min, 1% B; 8 min, 5% B; 15 min, 8% B; 20 min, 10% B; 25 min, 15% B; 35 min, 20% B; 40 min, 25% B; 50 min, 90% B; 53 min, 90% B; 58 min, 1% B; followed by re-equilibration at 1% B until 65 min.

Phenolic compounds were identified by comparing their retention times and UV–Vis absorption spectra with those of authentic analytical standards analyzed under identical chromatographic conditions. Quantification was performed using external calibration curves prepared from individual standard solutions. Calibration curves were established over an appropriate concentration range, and the linearity of each curve was verified prior to analysis.

Detection was performed simultaneously at multiple wavelengths selected according to the characteristic absorption maxima of different phenolic groups: 280 nm for hydroxybenzoic acids, 320 nm for hydroxycinnamic acids, and 365 nm for flavonols. Chromatograms and spectral data recorded within the range of 200–600 nm were additionally used to confirm compound identity.

Chromeleon™ Chromatography Data System software (version 6.8.1, Thermo Fisher Scientific, Waltham, MA, USA) was used for instrument control, data acquisition and chromatographic analysis. The concentrations of individual phenolic compounds were expressed as mg per 100 g (mg 100 g^−1^).

#### 3.2.8. Texture Analysis

The mechanical properties of the pasta, including the determination of the maximum cutting force, interpreted as firmness, and the energy, or work, required to cut the sample, were analyzed immediately after cooking. Measurements were performed using a TA-XT2 plus texture analyzer (Stable Micro Systems, Godalming, UK) equipped with a Warner–Bratzler adapter with a flat cutting blade. The blade speed during the test was 3 mm/s. Data acquisition and processing were carried out using Exponent software (version 4.0.13.0). Each test was performed in seven replicates. The two extreme values were rejected, and the arithmetic mean was calculated from the remaining five results.

#### 3.2.9. Color Analysis

The color of the pasta was determined instrumentally in the CIE Lab* color space. Light reflectance was measured using a CM-3500d spectrophotometer (Konica Minolta, Tokyo, Japan), with a 10° observer angle and a 30 mm measurement aperture. The samples were placed in Petri dishes with a diameter of 55 mm. The analysis included the determination of the following parameters:

L*, representing lightness, where 0 indicates black and 100 indicates white;

a*, representing the green-red color component, with negative values indicating green and positive values indicating red;

b*, representing the blue-yellow color component, with negative values indicating blue and positive values indicating yellow.

The total color difference in the enriched samples relative to the control variant (ΔE_00_) was calculated using the CIEDE2000 Formula (2). This approach accounts for the non-linearity of human visual perception by introducing corrections for lightness (ΔL′), chroma (ΔC′), and hue (ΔH′), as well as terms describing their mutual interactions. The use of the CIEDE2000 standard enables a more reliable and precise representation of visually perceived color differences.

(2) $$\Delta E_{00}=\sqrt{{\left(\frac{\Delta L^{\prime }}{k_{L}S_{L}}\right)}^{2}+{\left(\frac{\Delta C^{\prime }}{k_{C}S_{C}}\right)}^{2}+{\left(\frac{\Delta H^{\prime }}{k_{H}S_{H}}\right)}^{2}+R_{T}\left(\frac{\Delta C^{\prime }}{k_{C}S_{C}}\right)\left(\frac{\Delta H^{\prime }}{k_{H}S_{H}}\right)}$$

where

ΔL′—difference in lightness.
ΔC′—difference in chroma.
ΔH′—difference in hue.
SL, SC, SH—weighting functions for lightness, chroma, and hue, respectively.
RT—rotation term accounting for the interaction between hue and chroma.
kL, kC, kH—parametric correction factors.

Interpretation of color difference:

ΔE_00_ < 0.8—invisible difference, imperceptible to the human eye.
–ΔE_00_ = 0.8–1.5—very small difference, barely noticeable.
–ΔE_00_ = 1.5–3.0—small difference, noticeable but still acceptable.
–ΔE_00_ = 3.0–5.0—moderate difference, clearly visible.
–ΔE_00_ > 5.0—large difference, easily noticeable.

#### 3.2.10. Analysis of Cooking Properties

The swelling index (SI) was expressed as g water/g DM and defined as the amount of water absorbed by 1 g of pasta dry matter during thermal treatment.

Water absorption (WA) was expressed as g water/100 g product (3). This parameter described the amount of water absorbed by pasta during hydrothermal treatment, resulting from swelling and gelatinization of starch granules present in its structure. Water absorption was calculated according to the equation proposed by Tudorica et al. [95]:

(3) $$WA=\frac{a-b}{b}\times 100$$

where:

a—weight of pasta after cooking (g)

b—weight of pasta before cooking (g)

The weight increase after cooking (WIC) was expressed as the ratio of pasta weight after cooking to the weight of the product before cooking. This relative value describes the ability of the pasta matrix to increase in weight as a result of hydration and swelling of structural components during thermal treatment.

Cooking loss (CL) was determined according to the method described by Freire et al. [96], with minor modifications. Briefly, 10 g of pasta was cooked in 500 cm^3^ of boiling deionized water for the previously determined optimal cooking time (OCT). After cooking, the pasta was drained, and the cooking water was collected, transferred to pre-weighed porcelain crucibles, evaporated, and dried at 110 °C for 12 h. The dry residue remaining after drying was weighed and expressed as grams of dry matter lost per 100 g of raw pasta.

#### 3.2.11. Consumer Acceptance Evaluation

Consumer acceptability of the cooked pasta samples was assessed in accordance with the recommendations of PN-ISO 8589:1998 [97]. The study was conducted in accordance with the ethical principles set out in the Declaration of Helsinki [98], ensuring voluntary and informed participation of the panelists, protection of their privacy, and minimization of any potential discomfort associated with participation in the sensory evaluation.

The analysis was performed using a 9-point hedonic scale (Table 11), in which participants assigned scores from 1 to 9 to each variant. In the applied scale, a score of 1 indicated the lowest rating, corresponding to an extremely undesirable product, whereas a score of 9 indicated the highest rating, corresponding to an extremely desirable product. The obtained results made it possible to determine the level of sensory acceptance of the analyzed pasta samples and to compare them in terms of overall consumer evaluation.

#### 3.2.12. Statistical Analysis

The obtained results were subjected to statistical analysis using one-way analysis of variance (ANOVA). The significance of differences between mean values was determined using Duncan’s test at a significance level of *p* ≤ 0.05. The results were marked with letters indicating statistically homogeneous groups, with letter designations assigned from the lowest to the highest values. Values marked with the same letter or with a shared letter designation, e.g., ab, bc, or cd, did not differ significantly, whereas values marked with different letters, e.g., a, b, c, or d, differed significantly.

## 4. Conclusions

The present study demonstrates that extruded sugar beet pulp (ESBP) can be used as a functional ingredient in gluten-free pasta; however, its effects were strongly dependent on both the level of incorporation and the composition of the pasta matrix. The responses observed in conventional and high-protein formulations indicate that the nutritional and technological consequences of ESBP enrichment cannot be considered independently of the interactions between the additive and the other formulation components.

ESBP significantly modified the phenolic profile of the pasta. The highest apparent total phenolic content determined using the Folin–Ciocalteu assay was observed at the 15% addition level in both pasta types, whereas the non-Folin method indicated a significant enrichment at both 10% and 15% ESBP, with no further significant increase between these levels. HPLC-DAD analysis confirmed the enrichment of the pasta with specific phenolic acids. Hydroxybenzoic acids, including gallic, 4-hydroxybenzoic, and vanillic acids, predominated, while the hydroxycinnamic fraction included ferulic and *p*-coumaric acids. In conventional pasta containing 15% ESBP, the contents of 4-hydroxybenzoic and gallic acids increased by approximately 44% and 26%, respectively, and vanillic acid, which was not detected in the control, became measurable. In the high-protein formulation, even 5% ESBP increased the gallic acid content by approximately 48%. These results confirm that ESBP can increase the concentration and diversity of extractable phenolic acids in the final product, although the extent of this effect depends on the pasta matrix.

The incorporation of ESBP also affected the cooking properties of the pasta. Increases in the swelling index, water absorption, and weight increase after cooking indicated enhanced hydration and swelling, particularly in the conventional formulations. However, the high-protein pasta containing 15% ESBP exhibited a pronounced increase in cooking loss, indicating a reduced ability of the matrix to retain soluble components at the highest addition level. ESBP also caused progressive darkening and changes in the chromatic coordinates of the pasta. At the 10% and 15% addition levels, the calculated ΔE_00_ values indicated that the color differences would be readily perceptible. Despite these changes, ESBP did not markedly reduce pasta firmness, and in some formulations it increased cutting work, suggesting that moderate enrichment did not compromise the mechanical integrity of the product.

The consumer acceptance testing results showed that the highest ESBP level, particularly in high-protein pasta, reduced consumer acceptance, mainly because of lower ratings for taste, aroma, and overall acceptability. Therefore, although the 15% addition produced the greatest enrichment in selected phenolic compounds, it was also associated with less favorable cooking and sensory properties. Under the conditions of the present study, a 10% ESBP addition provided the most advantageous balance between phenolic enrichment, antioxidant potential, hydration-related cooking properties, texture, and sensory acceptance. This level may therefore be recommended as a practical starting point for the development of gluten-free pasta enriched with extruded sugar beet pulp, whereas higher addition levels may require further formulation optimization, particularly in high-protein matrices.

The present study has several limitations. It did not include a direct comparison between raw and extruded sugar beet pulp, and the assessment of bioactive compounds was based mainly on chemical and in vitro antioxidant assays, which do not directly reflect their gastrointestinal bioaccessibility or biological effects. Future studies should therefore investigate the influence of different extrusion conditions, evaluate phenolic bioaccessibility using simulated gastrointestinal digestion, and assess storage stability and consumer acceptance in larger and more diverse populations. Further optimization of the high-protein matrix is also recommended to reduce cooking losses and improve consumer acceptance at higher ESBP addition levels.

## Figures and Tables

**Table 1:** Nutritional composition and bioactive compound content of extruded sugar beet pulp (ESBP).

Parameter/Compound	Unit	Value
**Nutritional components**		
Dietary fiber	g/100 g DM	58.00 ± 0.10 *
Carbohydrates		21.30 ± 0.10
including sugars		18.00 ± 0.15
Protein		6.20 ± 0.05
Fat		1.00 ± 0.05
**Bioactive compounds and antioxidant activity**		
Total phenolic content with F.C.	mg catechin/100 g DM	151.04 ± 15.20
Total polyphenols without F.C.	mg catechin/100 g DM	59.10 ± 2.80
Phenolic acids	mg ferulic acid/100 g DM	18.16 ± 2.80
Flavonols	mg quercetin/100 g DM	12.00 ± 0.80
Flavonoids	mg rutin/100 g DM	23.90 ± 1.20
Anthocyanins	mg cyanidin-3-glucoside/100 g DM	ND ^1^
ABTS ^2^	mM Tx (Trolox)/g DM	3.84 ± 0.05
ABTS	mM Tx/kg DM	15.67 ± 0.23
DPPH ^3^	mg Tx/g DM	1.72 ± 0.03

^1^ ND—Not Detected, ^2^ ABTS—2,2′-azinobis(3-ethylbenzothiazoline-6-sulfonic acid) radical cation assay, ^3^ DPPH—2,2-diphenyl-1-picrylhydrazyl radical assay, * data are presented as mean ± SD.

**Table 2:** Content of UV-absorbing phenolic compounds in conventional and high-protein gluten-free pasta enriched with ESBP.

Sample	Total Phenolic with F.C. [mg Catechin/100 g DM]	Total Polyphenols Without F.C. [mg Catechin/100 g DM]
K_N *	94.3 ± 12.1 b **	49.2 ± 2.1 a
N_5%	79.4 ± 15.2 ab	50.3 ± 1.8 a
N_10%	56.2 ± 2.3 a	54.1 ± 2.2 b
N_15%	162.4 ± 24.3 c	55.4 ± 1.9 b
K_HP	248.3 ± 14.2 bc	26.1 ± 1.1 a
HP_5%	49.4 ± 7.2 a	29.3 ± 1.8 b
HP_10%	156.2 ± 17.4 ab	34.2 ± 1.2 c
HP_15%	364.3 ± 56.1 c	32.4 ± 1.1 c

* K_N—control sample of conventional gluten-free pasta without ESBP addition; N_5%—conventional gluten-free pasta containing 5% ESBP; N_10%—conventional gluten-free pasta containing 10% ESBP; N_15%—conventional gluten-free pasta containing 15% ESBP; K_HP—control sample of high-protein gluten-free pasta without ESBP addition; HP_5%—high-protein gluten-free pasta containing 5% ESBP; HP_10%—high-protein gluten-free pasta containing 10% ESBP; HP_15%—high-protein gluten-free pasta containing 15% ESBP, ** Values in the same column, designated by different letters (analyzed separately for each pasta type), are significantly different (*p* ≤ 0.05).

**Table 3:** Content of selected phenolic compound fractions in conventional and high-protein gluten-free pasta enriched with ESBP.

Sample	Phenolic Acids mg [Ferulic Acid/100 g DM]	Flavonols [mg Quercetin/100 g DM]	Flavonoids [mg Rutin/100 g DM]
K_N *	8.61 ± 1.03 ab **	5.53 ± 1.82 a	9.05 ± 1.10 a
N_5%	6.90 ± 2.72 a	2.70 ± 1.82 a	10.06 ± 1.09 ab
N_10%	10.72 ± 3.41 ab	5.54 ± 1.04 a	11.03 ± 2.02 b
N_15%	26.60 ± 3.70 c	5.21 ± 0.82 a	12.05 ± 1.06 b
K_HP	22.21 ± 2.04 c	20.91 ± 2.03 d	9.02 ± 1.10 a
HP_5%	0.91 ± 0.17 a	1.91 ± 0.43 a	11.04 ± 1.20 b
HP_10%	11.03 ± 0.30 b	8.10 ± 2.43 b	11.05 ± 2.06 b
HP_15%	24.84 ± 5.11 c	16.11 ± 1.62 c	11.05 ± 1.08 b

* K_N—control sample of conventional gluten-free pasta without ESBP addition; N_5%—conventional gluten-free pasta containing 5% ESBP; N_10%—conventional gluten-free pasta containing 10% ESBP; N_15%—conventional gluten-free pasta containing 15% ESBP; K_HP—control sample of high-protein gluten-free pasta without ESBP addition; HP_5%—high-protein gluten-free pasta containing 5% ESBP; HP_10%—high-protein gluten-free pasta containing 10% ESBP; HP_15%—high-protein gluten-free pasta containing 15% ESBP, ** Values in the same column, designated by different letters (analyzed separately for each pasta type), are significantly different (*p* ≤ 0.05).

**Table 4:** Antioxidant activity of conventional and high-protein gluten-free pasta enriched with ESBP.

Sample	DPPH [mg Tx/g DM]	ABTS [mM Tx/g DM]	ABTS [mM Tx/kg DM]
K_N *	1.63 ± 0.02 c **	ND ***	ND
N_5%	1.47 ± 0.02 b	0.51 ± 0.03 a	2.08 ± 0.12 a
N_10%	1.48 ± 0.01 b	1.46 ± 0.07 b	5.93 ± 0.28 b
N_15%	1.39 ± 0.02 a	1.98 ± 0.11 c	7.70 ± 0.35 c
K_HP	1.46 ± 0.03 a	1.17 ± 0.05 c	4.67 ± 0.22 c
HP_5%	1.45 ± 0.02 a	0.65 ± 0.04 a	2.65 ± 0.15 a
HP_10%	1.47 ± 0.01 a	0.60 ± 0.03 a	2.45 ± 0.14 a
HP_15%	1.52 ± 0.02 b	0.77 ± 0.04 b	3.05 ± 0.18 b

* K_N—control sample of conventional gluten-free pasta without ESBP addition; N_5%—conventional gluten-free pasta containing 5% ESBP; N_10%—conventional gluten-free pasta containing 10% ESBP; N_15%—conventional gluten-free pasta containing 15% ESBP; K_HP—control sample of high-protein gluten-free pasta without ESBP addition; HP_5%—high-protein gluten-free pasta containing 5% ESBP; HP_10%—high-protein gluten-free pasta containing 10% ESBP; HP_15%—high-protein gluten-free pasta containing 15% ESBP, ** Values in the same column, designated by different letters (analyzed separately for each pasta type), are significantly different (*p* ≤ 0.05), *** ND—Not Detected.

**Table 5:** Qualitative and Quantitative HPLC Analysis of Polyphenols in conventional and high-protein gluten-free pasta enriched with ESBP [mg/100 g D.M.].

Sample	Hydroxycinnamic Acids				Hydroxybenzoic Acids		
	*p*-Coumaric Acid	Ferulic Acid	Total	Gallic Acid	Vanillic Acid	4-Hydroxybenzoic Acid	Total
K_N *	nd	0.17 ± 0.00 a	0.17	0.49 ± 0.00 a	nd	0.16 ± 0.02 a	0.65
N_5%	0.10 ± 0.02 a **	0.17 ± 0.01 a	0.27	0.52 ± 0.03 a	0.08 ± 0.01 a	0.18 ± 0.01 a	0.78
N_10%	0.09 ± 0.01 a	0.17 ± 0.00 a	0.26	0.55 ± 0.01 a	0.09 ± 0.01 a	0.23 ± 0.00 b	0.87
N_15%	0.10 ± 0.00 a	0.18 ± 0.00 ab	0.28	0.62 ± 0.00 b	0.11 ± 0.00 b	0.23 ± 0.01 b	0.96
K_HP	0.08 ± 0.00 a	0.13 ± 0.00 a	0.21	0.88 ± 0.01 a	nd	0.28 ± 0.02 a	1.16
HP_5%	0.08 ± 0.00 a	0.14 ± 0.01 a	0.22	1.30 ± 0.00 b	0.06 ± 0.00 a	0.29 ± 0.03 a	1.65
HP_10%	0.10 ± 0.01 ab	0.16 ± 0.01 a	0.26	1.31 ± 0.03 b	0.09 ± 0.01 b	0.34 ± 0.02 a	1.74
HP_15%	0.09 ± 0.01 ab	0.18 ± 0.00 b	0.27	1.34 ± 0.00 b	0.08 ± 0.00 b	0.30 ± 0.00 a	1.72

* K_N—control sample of conventional gluten-free pasta without ESBP addition; N_5%—conventional gluten-free pasta containing 5% ESBP; N_10%—conventional gluten-free pasta containing 10% ESBP; N_15%—conventional gluten-free pasta containing 15% ESBP; K_HP—control sample of high-protein gluten-free pasta without ESBP addition; HP_5%—high-protein gluten-free pasta containing 5% ESBP; HP_10%—high-protein gluten-free pasta containing 10% ESBP; HP_15%—high-protein gluten-free pasta containing 15% ESBP, ** Values in the same column, designated by different letters (analyzed separately for each pasta type), are significantly different (*p* ≤ 0.05).

**Table 6:** Firmness and cutting work of conventional and high-protein gluten-free pasta samples enriched with ESBP.

Sample	Firmness (±SD) [N]	Work of Cutting (±SD) [N·s]
K_N *	3.16 ± 0.16 a **	9.20 ± 0.15 ab
N_5%	3.08 ± 0.24 a	9.15 ± 0.28 ab
N_10%	3.47 ± 0.28 a	9.37 ± 0.26 ab
N_15%	3.08 ± 0.21 a	9.15 ± 0.34 ab
K_HP	2.95 ± 0.04 a	8.98 ± 0.08 a
HP_5%	3.08 ± 0.27 a	9.06 ± 0.24 a
HP_10%	3.06 ± 0.22 a	9.14 ± 0.11 ab
HP_15%	3.11 ± 0.18 a	9.65 ± 0.40 b

* K_N—control sample of conventional gluten-free pasta without ESBP addition; N_5%—conventional gluten-free pasta containing 5% ESBP; N_10%—conventional gluten-free pasta containing 10% ESBP; N_15%—conventional gluten-free pasta containing 15% ESBP; K_HP—control sample of high-protein gluten-free pasta without ESBP addition; HP_5%—high-protein gluten-free pasta containing 5% ESBP; HP_10%—high-protein gluten-free pasta containing 10% ESBP; HP_15%—high-protein gluten-free pasta containing 15% ESBP, ** Values in the same column, designated by different letters (analyzed separately for each pasta type), are significantly different (*p* ≤ 0.05).

**Table 7:** Color parameters of raw and cooked conventional and high-protein gluten-free pasta enriched with ESBP.

	Sample	L*	a*	b*	ΔE_00_
RAW PASTA	K_N *	76.19 ± 0.80 c **	3.29 ± 0.27 b	27.69 ± 1.49 d	-
	N_5%	71.08 ± 0.15 b	3.10 ± 0.03 b	23.44 ± 0.11 c	4.28 ± 0.13 a
	N_10%	68.49 ± 0.36 a	2.41 ± 0.08 a	21.44 ± 0.17 b	6.53 ± 0.24 b
	N_15%	67.91 ± 0.56 a	2.26 ± 0.08 a	19.52 ± 0.57 a	7.43 ± 0.23 c
COOKED PASTA	K_N	70.80 ± 0.17 d	1.63 ± 0.08 a	25.15 ± 0.43 c	-
	N_5%	61.55 ± 0.90 c	2.54 ± 0.08 b	22.51 ± 0.55 b	7.68 ± 0.80 a
	N_10%	57.54 ± 0.75 b	2.53 ± 0.14 b	19.25 ± 0.24 a	11.49 ± 0.64 b
	N_15%	56.18 ± 0.26 a	2.82 ± 0.12 c	19.46 ± 0.35 a	12.69 ± 0.23 c
RAW PASTA	K_HP	68.53 ± 0.34 d	6.50 ± 0.11 d	26.70 ± 0.12 d	-
	HP_5%	66.75 ± 0.13 c	5.11 ± 0.09 c	24.57 ± 0.27 c	1.97 ± 0.08 a
	HP_10%	64.88 ± 0.23 b	4.47 ± 0.07 b	22.90 ± 0.20 b	3.70 ± 0.18 b
	HP_15%	63.66 ± 0.27 a	4.14 ± 0.12 a	21.55 ± 0.20 a	4.90 ± 0.13 c
COOKED PASTA	K_HP	61.80 ± 0.81 b	5.22 ± 0.22 c	21.39 ± 1.76 b	-
	HP_5%	56.46 ± 0.55 a	4.62 ± 0.25 b	19.68 ± 1.47 b	4.92 ± 0.39 a
	HP_10%	55.49 ± 0.97 a	4.29 ± 0.28 b	18.80 ± 1.77 b	5.95 ± 0.65 b
	HP_15%	56.34 ± 0.90 a	3.41 ± 0.19 a	14.99 ± 0.86 a	6.17 ± 0.40 b

L*—lightness; a*—red–green color coordinate; b*—yellow–blue color coordinate; ΔE_00_—total color difference calculated using the CIEDE2000 formula. * K_N—control sample of conventional gluten-free pasta without ESBP addition; N_5%—conventional gluten-free pasta containing 5% ESBP; N_10%—conventional gluten-free pasta containing 10% ESBP; N_15%—conventional gluten-free pasta containing 15% ESBP; K_HP—control sample of high-protein gluten-free pasta without ESBP addition; HP_5%—high-protein gluten-free pasta containing 5% ESBP; HP_10%—high-protein gluten-free pasta containing 10% ESBP; HP_15%—high-protein gluten-free pasta containing 15% ESBP, ** Values in the same column, designated by different letters (analyzed separately for each pasta type), are significantly different (*p* ≤ 0.05).

**Table 8:** Cooking properties of conventional and high-protein gluten-free pasta enriched with ESBP.

Sample	SI * [g Water/g DM]	WA [g Water/100 g]	WIC [Relative Value]	CL [g DM/100 g]
K_N **	1.43 ± 0.00 a ***	153.92 ± 0.00 a	2.54 ± 0.01 a	5.41 ± 1.20 ab
N_5%	1.47 ± 0.08 a	155.73 ± 0.71 b	2.56 ± 0.09 a	4.40 ± 1.33 a
N_10%	1.65 ± 0.01 b	176.06 ± 0.39 c	2.76 ± 0.02 b	6.84 ± 1.06 ab
N_15%	1.72 ± 0.03 b	183.89 ± 1.03 d	2.84 ± 0.02 b	7.82 ± 0.48 b
K_HP	1.30 ± 0.01 a	141.99 ± 0.80 a	2.16 ± 0.00 a	5.36 ± 0.12 a
HP_5%	1.41 ± 0.02 a	151.60 ± 0.52 b	2.27 ± 0.04 a	6.58 ± 0.95 a
HP_10%	1.44 ± 0.02 a	155.10 ± 0.96 c	2.30 ± 0.02 ab	8.19 ± 1.49 a
HP_15%	1.68 ± 0.17 b	174.96 ± 1.46 d	2.57 ± 0.22 b	15.26 ± 1.81 b

* SI—swelling index, WA—water absorption, WIC—weight increase after cooking, CL—cooking loss, ** K_N—control sample of conventional gluten-free pasta without ESBP addition; N_5%—conventional gluten-free pasta containing 5% ESBP; N_10%—conventional gluten-free pasta containing 10% ESBP; N_15%—conventional gluten-free pasta containing 15% ESBP; K_HP—control sample of high-protein gluten-free pasta without ESBP addition; HP_5%—high-protein gluten-free pasta containing 5% ESBP; HP_10%—high-protein gluten-free pasta containing 10% ESBP; HP_15%—high-protein gluten-free pasta containing 15% ESBP, *** Values in the same column, designated by different letters (analyzed separately for each pasta type), are significantly different (*p* ≤ 0.05).

**Table 9:** Consumer evaluation results of conventional and high-protein gluten-free pasta enriched with ESBP.

Sample	Appearance	Color	Aroma	Taste	Firmness	Overall Acceptability
K_N *	5.25 ± 0.42 a **	7.50 ± 0.77 b	6.75 ± 0.64 c	7.50 ± 0.56 c	4.50 ± 0.29 a	5.75 ± 0.29 bc
N_5%	6.00 ± 0.23 ab	6.25 ± 0.72 ab	6.00 ± 0.56 abc	5.25 ± 0.62 ab	5.75 ± 0.21 bc	6.00 ± 0.78 bc
N_10%	5.50 ± 0.70 a	5.50 ± 0.33 a	7.00 ± 0.31 c	5.00 ± 0.31 a	5.25 ± 0.38 ab	5.50 ± 0.51 bc
N_15%	6.00 ± 0.46 ab	6.25 ± 0.37 ab	6.00 ± 0.57 abc	4.75 ± 0.28 a	5.75 ± 0.38 bc	5.25 ± 0.42 ab
K_HP	5.75 ± 0.47 a	7.25 ± 0.67 b	6.25 ± 0.32 bc	6.50 ± 0.51 bc	5.75 ± 0.56 bc	6.50 ± 0.23 c
HP_5%	6.00 ± 0.56 ab	6.25 ± 0.30 ab	6.00 ± 0.24 abc	5.25 ± 0.77 ab	5.75 ± 0.78 bc	6.00 ± 0.69 bc
HP_10%	7.00 ± 0.38 b	5.50 ± 0.26 a	5.25 ± 0.61 b	5.50 ± 0.46 ab	6.50 ± 0.27 c	6.00 ± 0.50 bc
HP_15%	5.50 ± 0.22 a	5.25 ± 0.75 a	5.00 ± 0.36 a	5.25 ± 0.60 ab	5.00 ± 0.39 ab	4.25 ± 0.51 a

* K_N—control sample of conventional gluten-free pasta without ESBP addition; N_5%—conventional gluten-free pasta containing 5% ESBP; N_10%—conventional gluten-free pasta containing 10% ESBP; N_15%—conventional gluten-free pasta containing 15% ESBP; K_HP—control sample of high-protein gluten-free pasta without ESBP addition; HP_5%—high-protein gluten-free pasta containing 5% ESBP; HP_10%—high-protein gluten-free pasta containing 10% ESBP; HP_15%—high-protein gluten-free pasta containing 15% ESBP, ** Values in the same column, designated by different letters (analyzed separately for each pasta type), are significantly different (*p* ≤ 0.05).

**Table 10:** Formulations of the developed products.

Ingredient	K_N *	N_5%	N_10%	N_15%	K_HP	HP_5%	HP_10%	HP_15%
Rice flour [g]	150.0	142.5	135.0	127.5	120.0	114.0	108.0	102.0
Corn flour [g]	75.0	71.25	67.5	63.75	60.0	57.0	54.0	51.0
Potato flour [g]	45.0	42.75	40.5	38.25	36.0	34.2	32.4	30.6
Tapioca flour [g]	30.0	28.5	27.0	25.5	24.0	22.8	21.6	20.4
Pea protein isolate [g]	–	–	–	–	60.0	57.0	54.0	51.0
Extruded sugar beet pulp [g]	–	15.0	30.0	45.0	–	15.0	30.0	45.0
Eggs [pcs.]	1.0	1.0	1.0	1.0	1.0	1.0	1.0	1.0
Rapeseed oil [cm^3^]	15.0	15.0	15.0	15.0	15.0	15.0	15.0	15.0
Salt [g]	1.0	1.0	1.0	1.0	1.0	1.0	1.0	1.0
Water [cm^3^]	100.0	100.00	100.0	100.0	100.0	110.0	120.0	135.0
Guar gum [g]	1.8	1.8	1.8	1.8	1.8	1.8	1.8	1.8

* K_N—control sample of conventional gluten-free pasta without ESBP addition; N_5%—conventional gluten-free pasta containing 5% ESBP; N_10%—conventional gluten-free pasta containing 10% ESBP; N_15%—conventional gluten-free pasta containing 15% ESBP; K_HP—control sample of high-protein gluten-free pasta without ESBP addition; HP_5%—high-protein gluten-free pasta containing 5% ESBP; HP_10%—high-protein gluten-free pasta containing 10% ESBP; HP_15%—high-protein gluten-free pasta containing 15% ESBP.

**Table 11:** Consumer evaluation sheet for pasta.

Quality Attribute	1 Point	2 Point	3 Point	4 Point	5 Point	6 Point	7 Point	8 Point	9 Point
External appearance									
Color									
Aroma									
Taste									
Firmness									
Product attractiveness									

## Data Availability

The data presented in this study are available on request from the corresponding author.

## Abbreviations

The following abbreviations are used in this manuscript:

ESBP	Extruded Sugar Beet Pulp
TPC	Total Polyphenol Content
F.C.	Folin–Ciocalteu reagent
ABTS	2,2′-azino-bis(3-ethylbenzothiazoline-6-sulfonic acid)
DPPH	2,2-dphenyl-1-picrylhydrazyl
SI	Swelling Index
WA	Water Absorption
WPM	Mass increase coefficient
CL	Cooking Loss
OTC	Optimal Cooking Time
DM	Dry matter (formerly *DM*)
